# The Dissector’s Manual of Practical and Surgical Anatomy

**Published:** 1856-11

**Authors:** 


ART. IV.—*The Dissector's Manual of Practical and Surgical Anatomy.* By ERASMUS WILSON, F. R. S., Author of a "System of Human Anatomy." Illustrated with 154 Wood Engravings. Edited by WILLIAM HUNT, M. D., Demonstrator of Anatomy in the University of Pennsylvania. Philadelphia: BLANCHARD & LEA. 1856.

This is a third American edition of that valuable hand-book for the dissection-room, which has so nearly usurped the place of the time-honored Dublin Dissector. Lacking somewhat in that precision of description which belongs to the Dublin book, it is still a very excellent work for its purpose, and seems to be so fully appreciated by American students, that any elaborate notice from us is unnecessary.

For sale by PHINNEY & Co.

